# Healthy Lifestyle and Quality of Life in Post-Operative Colorectal Cancer Patients: A Five-Month Observational Study

**DOI:** 10.3390/nu16010068

**Published:** 2023-12-25

**Authors:** Yi-Chiu Li, Hsi-Hsien Hsu, Shu-Ping Yang, Gwo-Chi Hu, Hui-Mei Wang, Wen-Chien Huang, Tsae-Jyy Wang

**Affiliations:** 1 Division of Colorectal, Department of Surgery, MacKay Memorial Hospital, Taipei City 10449, Taiwan; liyichiu99np@gmail.com (Y.-C.L.); hsu5936@ms3.hinet.net (H.-H.H.); 2 MacKay Junior College of Medicine Nursing and Management, Taipei City 11260, Taiwan; 3 Department of Nursing, Cathay General Hospital, Taipei City 10630, Taiwan; cgh251699@cgh.org.tw (S.-P.Y.); cgh259980@cgh.org.tw (H.-M.W.); 4 Department of Rehabilitation, MacKay Memorial Hospital, Taipei City 10449, Taiwan; kung527@gmail.com; 5 Department of Medicine, MacKay Medical College, New Taipei City 25245, Taiwan; 6 Department of Thoracic Surgery, MacKay Memorial Hospital, Taipei City 10449, Taiwan; 7 School of Nursing, National Taipei University of Nursing and Health Sciences, Taipei City 11219, Taiwan

**Keywords:** colorectal cancer, physical activity, fiber intake, sleep, obesity, lifestyles, health-related quality of life

## Abstract

Health-related quality of life (HRQOL) is an important indicator of treatment effectiveness. An unhealthy lifestyle can have a negative impact on quality of life. This study aimed to investigate changes in health-related lifestyle over time after surgery for colorectal cancer and their impact on HRQOL. Healthy lifestyle habits examined in this study included physical activity, smoking, alcohol consumption, fruit and vegetable intake, sleep, and obesity levels. An observational study design was used. A total of 75 post-operative colorectal cancer patients were recruited from two medical centers in Taiwan. Data were collected through structured questionnaires. Mean HRQOL scores at 1, 3, and 5 months after discharge were 102.5 (SD = 18.8), 102.9 (SD = 20.1), and 103.0 (SD = 18.9), respectively. A generalized estimating equation analysis showed that alcohol consumption (*p* = 0.009), fruit and vegetable intake (*p* = 0.020), physical activity (*p* = 0.023), sleep quality (*p* < 0.001), and obesity (*p* = 0.035) were important predictors of post-operative quality of life in patients with colorectal cancer. The impact of smoking on HRQOL did not reach statistical significance. Colorectal cancer patients tend to have better HRQOL after surgery if they stay physically active, eat enough fruits and vegetables, and sleep well.

## 1. Introduction

Colorectal cancer (CRC) ranks as the third most common cancer and is the second leading cause of cancer-related deaths globally [1]. The incidence rate of CRC is 35.4 per 100,000, with a mortality rate of 8.2 per 100,000. In Taiwan, CRC is the most prevalent form of cancer and is the third leading cause of cancer-related deaths, with a standardized incidence rate of 42.9 per 100,000 and a standardized mortality rate of 14.6 per 100,000 [2]. 

Unhealthy lifestyles such as obesity, lack of exercise, smoking, alcohol consumption, insufficient fruit and vegetable (fiber) intake, and poor sleep quality are risk factors for developing CRC [3,4,5,6,7] and may negatively affect treatment outcomes. A systematic literature review revealed that continuous supervised physical activity training can reduce the risk of CRC recurrence [8]. Adopting healthy lifestyle habits, such as not smoking and limiting alcohol intake, can reduce CRC-specific mortality by 35% [9]. The outcomes of a long-term follow-up study (median of 8 years) of CRC patients (*n* = 1575) highlighted that the hazard ratio and CRC-specific mortality of patients with a 5 g increase in daily fiber intake was 0.78 (95% CI, 0.65–0.93) and 0.86 (95% CI, 0.79–0.93), respectively [10]. Another study found that 70% of CRC patients (*n* = 1453) reported sleep problems, which had a negative impact on health outcomes [11]. Wele et al. [12] found that maintaining a normal body mass index (BMI), regular exercise, healthy eating habits, and minimizing alcohol and smoking slowed the progression of CRC. 

Based on the above literature, we can reliably infer that maintaining a healthy lifestyle can improve the treatment outcomes and increase the survival rates of CRC patients. However, there is limited research on the long-term monitoring of the post-operative health-related lifestyle of CRC patients.

Health-related quality of life (HRQOL) is an essential indicator of treatment effectiveness and a predictor of treatment trajectories for CRC patients [13]. Among long-term (median ≥ 7 years) CRC survivors (*n* = 1294), a 10-point increase in HRQOL scores reduced the risk of death by 24% (*p*  <  0.0001) [14]. Previous studies have found that nonmodifiable factors such as demographics, disease characteristics, and treatment characteristics affect the HRQOL of patients [14,15,16]. 

However, there is limited research on how the post-treatment health-related lifestyles of discharged CRC patients affect their HRQOL. Therefore, the purpose of this study is to explore how the post-treatment health-related lifestyles of CRC patients change over time and their impact on HRQOL. The health-related lifestyles investigated include physical activity, smoking, alcohol consumption, fruit and vegetable intake, sleep quality, and obesity.

## 2. Materials and Methods

### 2.1. Study Design

This study adopts an observational research design, focusing on post-treatment CRC patients. Data were collected through a questionnaire survey completed at one month, three months, and five months after the completion of treatment. This study was approved by the target hospital’s Institutional Review Board.

### 2.2. Sample and Setting

Convenience sampling was used to recruit CRC patients who had completed treatment and were discharged from the colorectal surgery wards of two medical centers in northern Taiwan. Patients who met the following eligibility criteria were recruited.

The inclusion criteria were as follows: (1) histologically diagnosed with primary colon or rectal cancer, (2) cancer stage I–II (nonmetastatic), (3) underwent curative tumor resection surgery, and (4) aged 20 years and older. The exclusion criteria were as follows: (1) poor physical condition (Eastern Cooperative Oncology Group [ECOG] performance score greater than or equal to 3), (2) severe cognitive impairment (Short Orientation Memory Cognitive Test score greater than or equal to 20), (3) diagnosed with severe mental illness by a physician, (4) physician-predicted life expectancy of less than 3 months, (5) prior history of cancer, or (6) inability to communicate verbally or in writing.

### 2.3. Data Collection and Study Instruments

Data in the self-administered questionnaires were collected by the researchers involved in this study and included demographics, smoking status, alcohol consumption status, physical activity, fruit and vegetable intake, sleep quality, and CRC-related quality of life. The researchers also extracted disease and treatment characteristics from the participants’ medical records, including pathological diagnosis, cancer stage, surgical method, adjuvant treatment, and comorbidities. These data were used to calculate the Charlson Comorbidity Index (CCI) [17].

The International Physical Activity Questionnaire-Short Form (IPAQ-SF) was used to measure physical activity [18]. This questionnaire assesses the time spent performing physical activity at various intensities (walking, moderate exercise, and vigorous exercise) over the past week. The metabolic equivalent of task (MET) minutes, which represent the total energy expenditure from physical activity, were calculated by multiplying the time spent at each intensity level by their respective MET values. For health purposes, adults should engage in at least 600 MET minutes of physical activity per week, equivalent to 150 minutes of brisk walking or 75 minutes of running per week [19]. The Chinese version of the IPAQ-SF used in this study demonstrated good validity and reliability [20].

The All-Day version of the Eating at America’s Table Study instrument was used to assess fruit and vegetable intake over the past month [21]. This questionnaire comprises ten questions regarding the quantity and frequency of fruit and vegetable consumption. The scale score is calculated by multiplying the quantity score and the frequency score, with higher scores indicating greater fruit and vegetable intake. This questionnaire is significantly correlated with 24-hour logged intake (*n* = 462) and has a coefficient of 0.66 for men and 0.51 for women [21].

The Pittsburgh Sleep Quality Index (PSQI) was used to assess sleep quality over the past month. The questionnaire comprises ten questions covering subjective sleep quality, sleep latency, sleep duration, sleep efficiency, sleep disturbances, use of sleep medications, and daytime dysfunction. The questions are scored on a four-point Likert scale. The total score, ranging from 0 to 21, indicates sleep quality, with higher scores (>5) indicating poor sleep quality. The PSQI has demonstrated good internal consistency and reliability in the past [22]. In this study, the Cronbach’s alpha coefficient for the PSQI was 0.62.

The Functional Assessment of Cancer Therapy-Colorectal (FACT-C) was used to assess CRC-related quality of life over the past week [23]. The scale consists of 36 items that assess physical, social/family, emotional, and functional well-being in CRC, as well as symptoms and treatment-related side effects. Each item is rated on a five-point Likert scale. Total scores on this scale range from 0 to 136. Higher scores indicate better quality of life. The FACT-C has demonstrated good reliability and validity in the past. The results of factor analysis supported the hypothesized dimensionality and confirmed the construct validity [24,25]. The Cronbach’s alpha coefficients for CRC groups ranged from 0.91 to 0.97 [25]. The Pearson correlation coefficients between the Traditional Chinese version of FACT-C and QLQ-C30/CR38 as well as SF-12v2 were greater than 0.4, indicating good concurrent validity [26]. The Cronbach’s alpha coefficient in this study was 0.93.

### 2.4. Statistical Analysis

Statistical analysis was conducted using SPSS 22.0 for Windows. Descriptive statistics were used to describe the central tendency and dispersion of the variables. Lifestyle variables (physical activity, fruit and vegetable intake, sleep quality, and obesity) were further categorized according to compliance versus noncompliance with health recommendations. Chi-square analysis was used to analyze differences in HRQOL between patients who met health recommendations and those who did not meet recommendations for each lifestyle variable. Generalized estimated equation (GEE) models were used to examine longitudinal associations of physical activity, fruit and vegetable intake, sleep quality, and obesity with HRQOL, while correcting for sociodemographic and clinical factors. 

## 3. Results

### 3.1. Demographics and Clinical Characteristics

During the recruitment period, 75 post-operative and discharged CRC patients who potentially met the sampling criteria were approached for recruitment. Among them, four individuals declined to participate in the study. In total, 70 individuals participated in this research. Three participants withdrew from the study in the third month due to a lack of interest or inability to cooperate, and one participant withdrew in the fifth month for similar reasons. Data from all 70 participants were included in the analysis. 

The average age of the participants was 61.0 years (SD = 13.2). Among them, 38 (54.3%) were male, and the majority had at least a college-level education (*n* = 26, 37.1%). Most of the participants were married (*n* = 48, 68.6%), were currently unemployed (*n* = 40, 57.2%), and had just enough financial resources (*n* = 42, 60.0%). Further, 21 participants were in stage I (30.0%), 25 were in stage IIA (35.7%), 4 were in stage IIB (5.7%), and 20 were in stage IIC (28.6%). Of the 70 participants, 38 underwent laparoscopic surgery (54.3%), while 32 underwent open surgery (45.7%). Twenty participants (28.6%) received post-operative intravenous (IV) chemotherapy, and 17 (24.3%) received post-operative oral chemotherapy. The average CCI was 0.38 (SD = 0.74). Regarding lifestyle factors, 11 participants (15.7%) reported that they consumed alcohol regularly, while 6 (8.6%) reported that they had quit. Seven participants (10.0%) reported that they smoked regularly, eight (11.4%) reported that they had quit, and the remaining participants reported that they were nonsmokers (Table 1).

### 3.2. Physical Activity, Fruit and Vegetable Intake, Sleep Quality, and Obesity

The average weekly physical activity of the participants after discharge was 1085.8 METs (SD = 1605.8) at one month, 2213.0 METs (SD = 2758.8) at three months, and 2592.9 METs (SD = 3405.5) at five months. A GEE analysis showed a significant time effect, with physical activity at three months after discharge being significantly higher than that at one month (B = 1122.2, 95% CI: 482.8~1761.7) and that at five months being significantly higher than that at one month (B = 1507.7, 95% CI: 794.9~2220.5; Table 2). These results indicate an increase in physical activity over time. The proportion of participants meeting the WHO recommendation of at least 600 METs per week for physical activity was 54.3% at one month, 67.6% at three months, and 79.2% at five months. A chi-square analysis showed a significant difference in the number of participants meeting the WHO recommendation at each time point (X^2^ = 7.8, *p* = 0.02), with more participants meeting the recommendation at three and five months (Table 3).

The average daily intake of fruits and vegetables was 4.9 servings (SD = 3.5) at one month, 6.2 servings (SD = 4.5) at three months, and 6.2 servings (SD = 4.5) at five months. A GEE analysis showed a significant effect of time, with fruit and vegetable intake at three months being significantly higher than that at one month (B = 1.33, 95% CI: 0.06~2.6; Table 2). The proportion of participants consuming at least five servings of fruits and vegetables per day was 45.7% at one month, 57.4% at three months, and 56.6% at five months. A chi-square analysis showed no significant difference in the number of participants meeting the five-a-day recommendation at each time point (X^2^ = 1.9, *p* = 0.378), indicating that most CRC patients consumed fewer than five servings of fruits and vegetables daily (Table 3). Although fruit and vegetable intake significantly increased from one month to three months after discharge, the number of individuals meeting the recommendation did not differ significantly.

The participants had an average sleep quality score of 7.3 (SD = 3.5) at one month, 7.0 (SD = 3.6) at three months, and 6.9 (SD = 3.5) at five months. A GEE analysis showed no significant time effect (Table 2), indicating that sleep quality did not change significantly over time. Further analysis of poor sleep quality (PSQI total score >5) at each time point revealed that 70.0% of patients experienced poor sleep quality at one month, 60.3% at three months, and 60.4% at five months. A chi-square analysis showed no significant difference in the number of individuals with poor sleep quality at each time point (X^2^ = 1.46, *p* = 0.483), indicating that most of the CRC patients experienced poor sleep quality that did not significantly improve over time (Table 3).

The average BMI of the participants after discharge was 24.1 kg/m^2^ (SD = 3.1) at one month, 24.4 kg/m^2^ (SD = 3.2) at three months, and 24.6 kg/m^2^ (SD = 3.4) at five months. A GEE analysis showed a significant effect of time, with BMI at three months being significantly higher than that at one month (B = 0.29, 95% CI: 0.28~0.30) and that at five months being significantly higher than that at one month (B = 0.51, 95% CI: 0.49~0.52) (Table 2). These results indicate an increase in BMI over time. The participants were categorized into the following groups based on their BMI: underweight (BMI < 18.5), healthy weight (18.5 ≤ BMI < 24), overweight (24 ≤ BMI < 27), mild obesity (27 ≤ BMI < 30), moderate obesity (30 ≤ BMI < 35), and severe obesity (35 ≤ BMI). At one month after discharge, 49.3% were in the healthy weight category, 31.3% were overweight, 14.9% had mild obesity, and 4.5% had moderate obesity. At three months after discharge, 44.6% were in the healthy weight category, 35.4% were overweight, 15.4% had mild obesity, and 4.6% had moderate obesity. At five months after discharge, 48.1% were in the healthy weight category, 25.0% were overweight, 19.2% had mild obesity, and 7.7% had moderate obesity. A chi-square analysis showed no significant difference in the distribution of different BMI categories at each time point (X^2^ = 2.20, *p* = 0.900; Table 3).

### 3.3. Impact of Physical Activity, Fruit and Vegetable Intake, Sleep Quality, and Obesity on HRQOL

The average HRQOL score of the participants after discharge was 102.5 (SD = 18.8) at one month, 102.9 (SD = 20.1) at three months, and 103.0 (SD = 18.9) at five months. A GEE analysis showed no significant time effect (Table 2), indicating that HRQOL did not change significantly over time. The FACT-C total score can range from 0 to 136, with higher scores indicating better quality of life. At each time point, the participants had an average FACT-C score of approximately 100, suggesting that the CRC patients had relatively good HRQOL after discharge. However, a portion of the participants had poor HRQOL, with the lowest 25% of participants scoring between 47–93 at one month, 51–93 at three months, and 56–89 at five months.

GEE analyses were conducted to assess the impact of the different lifestyle factors (alcohol consumption, physical activity, fruit and vegetable intake, sleep quality, and obesity) on HRQOL under controlled sociodemographic and clinical factors. The GEE model included time, sociodemographic factors (age, education level, marital status, employment status, and economic status), clinical factors (CCI severity, cancer staging, surgical method, and adjuvant treatment), smoking status, drinking habits, physical activity, fruit and vegetable intake, sleep quality, and obesity. Because of the high correlation between cancer stage and adjuvant treatment, only adjuvant treatment was included in the GEE model to avoid multicollinearity. The results showed that alcohol consumption habits (Wald X^2^ = 6.75, *p* = 0.009), physical activity (Wald X^2^ = 5.17, *p* = 0.023), fruit and vegetable intake (Wald X^2^ = 5.43, *p* = 0.020), sleep quality (Wald X^2^ = 22.16, *p* < 0.001), and obesity (Wald X^2^ = 4.44, *p* = 0.035) significantly affected HRQOL after controlling for time, demographics, and clinical conditions. Specifically, individuals who quit drinking had a lower HRQOL compared to those who did not consume alcohol (B = −13.69, 95% CI: −24.01~−3.36). Those who engaged in physical activity of at least 600 METs per week had a higher HRQOL compared to those who did not reach 600 METs (B = 5.45, 95% CI: 0.75~10.15). Participants who consumed at least five servings of fruits and vegetables per day had a higher HRQOL compared to those who did not (B = 5.88, 95% CI: 0.93~10.82), and participants with good sleep quality had a higher HRQOL compared to those with poor sleep quality (B = 16.08, 95% CI: 9.38~22.77). Lastly, moderately obese participants had a higher HRQOL compared to those with normal body weight (B = 9.99, 95% CI: 0.70~19.28). Interestingly, smoking habits did not have a statistically significant impact on HRQOL (Table 4).

## 4. Discussion

The results of this study showed that overall, the post-operative HRQOL of the discharged CRC patients was at an acceptable level and remained relatively stable over time [27]. HRQOL was measured using the FACT-C scale, which produces a possible score ranging from 0 to 136. The average scores of the participants at one, three, and five months after discharge were 102.5, 102.9, and 103.0, respectively. Similar findings were reported by Valeikaite et al. [28] in their study of long-term CRC patients (*n* = 88). The researchers of that study observed an improvement in the quality of life within two years after surgery, which was sustained for up to six years, as indicated by the QLQ C-30 scale (60; 69.5; 65.33; *p* = 0.06). Similarly, Al-Shandudi et al. [29] found that most CRC survivors (*n* = 118) had a good HRQOL, with an average QLQ C-30 score of 81.7 (out of 100).

Our study’s results support the findings in previous studies [30] that alcohol consumption, physical activity, fruit and vegetable intake, sleep quality, and obesity are important factors that influence the HRQOL of post-operative colorectal cancer patients. Specifically, individuals who did not drink, engaged in physical activity of at least 600 METs per week, consumed at least five servings of fruits and vegetables per day, had good sleep quality, and were moderately obese reported a higher HRQOL. These results support the findings in previous studies.

We found no difference in the HRQOL between drinkers and nondrinkers, but patients who had quit drinking had a lower HRQOL compared to those who did not drink. Previous studies have reported inconsistent results regarding the association between alcohol consumption and HRQOL in CRC survivors. A longitudinal study [6] investigated patients with colorectal cancer (*n* = 445) 24 months after diagnosis and found that those who drank more alcohol per week had a better HRQOL. Another longitudinal study examining female CRC survivors up to 20 years post-diagnosis found that anxiety was more associated with alcohol usage during follow-up [31]. On the other hand, a cross-sectional study of 155 CRC survivors 2–10 years after diagnosis showed that heavy drinking was associated with poorer physical functioning and increased fatigue [32]. These inconsistent findings may be due to differences in the study design and populations. Instead of collecting data on the amount of alcohol consumed [6], we only asked participants to indicate whether they are currently a regular drinker, had quit drinking, or were not a drinker, which may not be specific enough to conduct a more precise analysis. The impact of alcohol consumption on HRQOL in CRC patients warrants further study.

In the present study, the effect of smoking on HRQOL failed to reach statistical significance, possibly due to the relatively small number of smokers observed (seven current smokers [10.0%] and eight former smokers [11.4%]) and the follow-up period only being 9 months. Therefore, previous findings of lower HRQOL in smokers [33,34] cannot be confirmed by the results of the present study. A larger sample size and a longer follow-up period may have greater statistical power to detect the effects of smoking on HRQOL.

Our study indicated that CRC patients who engaged in 600 METs of physical activity per week (equivalent to 150 minutes of moderate intensity exercise per week) had a significantly higher HRQOL than those who did not achieve 600 METs. This finding supports the importance of maintaining appropriate levels of physical activity for post-discharge recovery and HRQOL. Brown et al. [35] conducted a small randomized controlled trial (RCT) involving CRC survivors within three years of treatment and found that engaging in 150 minutes of moderate intensity physical activity per week (*n* = 14) led to improved FACT-C scores compared to the control group (*n* = 13). Another systematic review of long-term CRC survivors (≥5 years) showed a positive correlation between moderate to high intensity physical activity and higher HRQOL, although most of the reviewed studies were cross-sectional [36]. A notable proportion of CRC patients in this study maintained regular physical activity. In contrast to the study of Rodriguez et al. [37], who observed that only 40.4% of the long-term CRC survivors (*n* = 593; ≥5 years) engaged in 150 minutes of physical activity or more per week, 54.3% of the participants in this study met the WHO-recommended 150 minutes per week one month after discharge, and this percentage increased to 79.2% by the fifth month. Unlike previous cross-sectional studies, this research represents a prospective long-term follow-up study of CRC patients after discharge.

Our study results revealed that CRC patients who met the daily recommendation of consuming five or more servings of fruits and vegetables (equivalent to approximately 25–35 grams of dietary fiber intake per day) had a significantly higher HRQOL compared to those who did not meet this recommendation. This supports the importance of maintaining adequate fruit and vegetable intake for post-discharge recovery and HRQOL [30]. Kenkhuis et al. [38] examined CRC survivors with stage I–III CRC in the Netherlands (*n* = 459) and also found that increasing fruit and vegetable intake by 100 grams per day and dietary fiber intake by 10 grams per day during the two years after CRC treatment was associated with improved physical function, role function, and HRQOL. 

It is worth noting that in the present study, the average daily fruit and vegetable intake of the participants in the initial month after discharge was averaged at 4.9 servings and was insufficient. This may be related to the adoption of a low-residue diet immediately after discharge. Nevertheless, by three months after discharge, the average daily fruit and vegetable intake increased significantly to 6.2 servings and remained at this level at five months post-discharge. On average, the participants met the recommendation of five servings of fruits and vegetables per day from three months post-discharge onwards, but a proportion of participants (54.3%, 42.6%, and 43.4% at one, three, and five months, respectively) did not meet this recommendation. The total fiber intake of the participants five months after discharge ranged from 24 to 31 grams, which coincided with the findings of Song et al. [10], who observed a similar distribution of total fiber intake among CRC patients six months after treatment (ranging from 14 grams in quartile 1 to 30 grams in quartile 4). Therefore, it is recommended that patients continue to increase their fiber intake after three months post-discharge.

We found that participants with good sleep quality have a higher HRQOL compared to those with poor sleep quality (PSQI > 5). This finding supports previous research highlighting the importance of sleep quality in the post-operative recovery and HRQOL of CRC patients [11]. Al-Shandudi et al. [29] found that insomnia was one of the three most distressing symptoms for CRC survivors (*n* = 118), and the severity of insomnia symptoms was associated with lower HRQOL. The average sleep quality scores of the participants in the present study were 7.3, 7.0, and 6.9 at one, three, and five months post-discharge, respectively. Furthermore, the proportion of participants with poor sleep quality was 70.0%, 60.3%, and 60.4% at these respective time points. These results indicate that not only did most CRC patients experience poor sleep quality after discharge, but also that their sleep quality did not improve significantly over time. This finding underscores the importance of monitoring and addressing issues related to sleep quality in this patient population.

Compared to previous findings that suggested that obesity (BMI > 30 kg/m^2^) is associated with lower physical function in CRC survivors [39], the findings of the present study showed a significant increase in BMI over time, and that individuals with moderate obesity had a higher HRQOL compared to those with normal weight. This may be attributed to CRC survivors increasing their physical activity to maintain an active lifestyle during recovery, which requires the expenditure of more calories, and the gradual transition from fasting to a normal diet with adequate caloric intake. Sufficient nutrition and the healthy expenditure of energy through extended physical activity contribute positively to physical function recovery and HRQOL. 

The main strength is that rather than using a cross-sectional study design, we used repeated measures and followed participants for five months to monitor changes in health-related lifestyle and HRQOL in patients with colorectal cancer after surgery. Additionally, the health-related lifestyle factors were also widely documented and repeatedly measured, including alcohol consumption, smoking, physical activity, fruit and vegetable intake, sleep quality, and obesity. By doing so, we were able to examine the impact of all health-related lifestyle behaviors on HRQOL simultaneously and over time.

This study has several limitations. First, this study used convenience sampling to limit recruitment to two medical centers in northern Taiwan. This may lead to sampling bias and a lack of diversity. Therefore, the results may not be generalized to the broader population of CRC patients. Future research with larger geographic coverage and random sampling methods will increase the generalizability of the findings. A second limitation is the use of self-administered questionnaires, which can be subject to memory limitations. Moreover, lengthy questionnaires containing multiple items may be affected by participant fatigue, which may affect measurement accuracy. Using objective measurement tools to assess fruit and vegetable intake, physical activity, and sleep quality may provide a more accurate assessment. Third, this study only assessed HRQOL at three time points after treatment completion (during a 5-month follow-up period), which may not reflect the long-term impact of CRC on patients’ quality of life. The effects of the variables investigated may differ during follow-up periods of one year or more. Lastly, exploring other factors that may influence HRQOL, such as social support or psychological well-being, may also influence the reported results.

## 5. Conclusions

The results of this study show that physical activity in post-operative CRC patients gradually increased over time, with 79.2% of participants meeting the recommended level of 600 METs per week within five months of discharge. However, fruit and vegetable intake did not increase significantly, with only 56.6% of study participants meeting the recommended intake of five servings per day within five months of discharge. Additionally, sleep quality remained poor after discharge, with more than 60% of study participants reporting PSQI scores above five at every time point. Overall, post-operative HRQOL of CRC patients is good and relatively stable after discharge. Alcohol consumption, physical activity, fruit and vegetable intake, sleep quality, and obesity are significant predictive factors of post-discharge HRQOL in patients undergoing colorectal cancer surgery. Under controlled demographic and clinical conditions, participants who abstained from alcohol, did not meet the recommended physical activity level of 600 MET per week, did not consume five or more servings of fruits and vegetables per day, and had poor sleep quality were identified as a high-risk group for having a poorer HRQOL. These findings highlight the importance of promoting healthy lifestyles in patients with CRC after discharge from hospital. Specifically, the focus should be on increasing fruit and vegetable intake and improving sleep quality. In addition, patients who have quit drinking should be continuously evaluated and monitored to improve their HRQOL.

## Figures and Tables

**Table 1:** Participants’ demographics and clinical characteristics (*n* = 70).

Variables	*n*	%	
Sex			
Male	38	54.3	
Female	32	45.7	
Education level			
Primary school and below	19	27.2	
Middle and high school	25	35.7	
College and above	26	37.1	
Marital status			
Married	46	68.2	
Divorce or widow	15	21.4	
Single	7	10.0	
Working status			
Full time	20	28.6	
Part time	5	7.1	
Others	5	7.1	
None	40	57.2	
Financial status			
Affluent	4	5.7	
Surplus	16	22.9	
Just enough	42	60.0	
Not enough income	8	11.4	
Cancer stage			
I	21	30.0	
IIA	25	35.7	
IIB	4	5.7	
IIC	20	28.6	
Types of surgery			
Laparotomy	32	45.7	
Laparoscopy	38	54.3	
Adjuvant therapy			
IV chemotherapy	20	28.6	
Oral chemotherapy	17	24.3	
None	33	47.1	
Drinking			
Yes	11	15.7	
Quit	6	8.6	
No	53	75.7	
Smoking			
Yes	7	10.0	
Quit	8	11.4	
No	55	78.6	
	Mean	SD	Range
Age	61.0	13.2	30–87
Charlson Comorbidity Index	0.38	0.74	0–2
Number of outpatient visits	6.0	1.4	3–10
Number of nutritional consultations	3.6	0.8	3–6

**Table 2:** Changes in physical activity, fiber intake, sleep quality, and health-related quality of life over time in patients with colorectal cancer (*n* = 70).

Variable	Time	*n*	Mean	SD	Range	*B*	95%CI		Wald	*p*
							Lower	Upper	*X* ^2^	
Physical activity	T3	53	2592.9	3405.5	0–16,506	1507.7	794.9	2220.5	17.2	<0.001
	T2	68	2213.0	2758.8	0–13,092	1122.2	482.8	1761.7	11.8	0.001
	T1	70	1085.8	1605.8	0–9219	0a				
Fiber intake	T3	53	6.2	4.5	0.5–27.9	1.29	−0.11	2.68	3.27	0.071
	T2	68	6.2	4.5	0.3–32.6	1.33	0.06	2.60	4.19	0.041
	T1	70	4.9	3.5	0–16	0a				
Sleep quality	T3	53	6.9	3.5	1–16	−0.65	−1.4	0.07	3.17	0.075
	T2	68	7.0	3.6	1–16	−0.29	−0.94	0.37	0.74	0.390
	T1	70	7.3	3.5	1–17	0a				
BMI	T3	52	24.6	3.4	18.6–33.2	0.51	0.49	0.52	6499	<0.001
	T2	65	24.4	3.2	18.7–32.3	0.29	0.28	0.30	2481	<0.001
	T1	67	24.1	3.1	18.7–30.8	0 a				
HRQOL	T3	53	103.0	18.9	56–128	0.47	−3.78	4.71	0.05	0.827
	T2	68	102.9	20.1	51–132	0.32	−3.57	4.21	0.03	0.872
	T1	70	102.5	18.8	47–130	0a				

Note. Changes in lifestyle variables and HRQOL over time were analyzed using generalized estimating equations (GEE) with an AR(1) correlation structure.

**Table 3:** Number of patients who meet the recommendations in physical activity, fiber intake, andgood sleep quality at different time points (*n* = 70).

Variables	Time	Meeting the Recommendation				Not Meeting the Recommendation				X2	*p*
		*n*		%		*n*		%			
Physical activity (>600 METs/wk)	T3	42		79.2		11		20.8		7.8	0.02
	T2	46		67.6		22		32.4			
	T1	38		54.3		32		45.7			
Fiber intake (>5/day)	T3	30		56.6		23		43.4		1.9	0.378
	T2	39		57.4		29		42.6			
	T1	32		45.7		38		54.3			
		Good				Not good					
		*n*		%		*n*		%			
Sleep quality (<5)	T3	21		39.6		32		60.4		1.46	0.483
	T2	27		39.7		41		60.3			
	T1	21		30.0		49		70.0			
		Normal weight		Overweight		Slight obesity		Moderate obesity			
BMI category		*n*	%	*n*	%	*n*	%	*n*	%	2.2	0.900
	T3	25	48.1	13	25.0	10	19.2	4	7.7		
	T2	29	44.6	23	35.4	10	15.4	3	4.6		
	T1	33	49.3	21	31.3	10	14.9	3	4.5		

**Table 4:** Parameters of the generalized linear model for exploring the potential influences of the study variables on health-related quality of life change over time (*n* = 70).

Variables	B	SE	95% CI		Wald X^2^	*p* Value
			Lower	Upper		
T3 vs. T1	−2.47	2.18	−6.75	1.81	1.28	0.258
T2 vs. T1	−2.55	2.08	−6.64	1.53	1.51	0.220
Age	−0.01	0.19	−0.39	0.36	0.01	0.944
Female vs. male	0.84	4.36	−7.71	9.38	0.04	0.848
College vs. primary school	9.38	5.20	−0.82	19.57	3.25	0.071
High–middle vs. primary school	16.57	5.49	5.80	27.33	9.10	0.003
Married vs. single	18.64	6.91	5.12	32.17	7.30	0.007
Divorced vs. single	14.09	5.08	4.13	24.05	7.69	0.006
Full time vs. none	10.08	6.66	−2.970	23.12	2.291	0.130
Part time vs. none	−4.95	4.67	−14.11	4.22	1.12	0.290
Others vs. none	−4.98	5.27	−15.31	5.35	0.89	0.345
Affluent vs. not enough	−2.61	5.74	−13.85	8.64	0.21	0.649
Surplus vs. not enough	2.92	5.88	−8.59	14.44	0.25	0.619
Just enough vs. not enough	−5.35	4.75	−14.65	3.96	1.27	0.260
CCI	−2.01	3.40	−8.67	4.64	0.35	0.55
Laparotomy vs. laparoscopy	1.10	3.26	−5.30	7.49	0.11	0.737
IV chemotherapy vs. none	−5.15	5.08	−15.11	4.82	1.02	0.31
Oral chemotherapy vs. none	2.15	4.96	−7.57	11.87	0.19	0.66
Quit vs. no drinking	−13.69	5.27	−24.01	−3.36	6.75	0.009
Yes vs. no drinking	5.60	5.28	−4.74	15.95	1.13	0.289
Yes vs. no smoking	5.38	4.78	−3.99	14.75	1.27	0.261
Quit vs. no smoking	−3.20	5.51	−13.99	7.59	0.338	0.561
Physical activity (met vs. not met)	5.45	2.40	0.75	10.15	5.17	0.023
Fiber intake (met vs. not met)	5.88	2.52	0.93	10.82	5.43	0.020
Sleep (good vs. not good)	16.08	3.42	9.38	22.77	22.16	<0.001
Moderate obesity vs. normal	9.99	4.74	0.70	19.28	4.44	0.035
Slight obesity vs. normal	8.08	5.11	−1.95	18.10	2.50	0.114
Overweight vs. normal	1.74	3.54	−5.20	8.68	0.24	0.623

Note. Using generalized estimation equations with an AR(1) correlation structure.

## Data Availability

The data are not publicly available because they contain information that could compromise the privacy of study participants. However, data supporting the results of this study are available from the corresponding author, T.-J.W., upon reasonable request.

## References

[1] Morgan E., Arnold M., Gini A., Lorenzoni V., Cabasag C.J., Laversanne M., Vignat J., Ferlay J., Murphy N., Bray F. (2023). Global burden of colorectal cancer in 2020 and 2040: Incidence and mortality estimates from GLOBOCAN. Gut.

[2] Taiwan Health Promotion Administration (2020). Cancer Registration Report. https://www.hpa.gov.tw/Pages/List.aspx?nodeid=269.

[3] Li H., Chen X., Hoffmeister M., Brenner H. (2023). Associations of smoking with early-and late-onset colorectal cancer. JNCI Cancer Spectr..

[4] Ma Y., Hu M., Zhou L., Ling S., Li Y., Kong B., Huang P. (2018). Dietary fiber intake and risks of proximal and distal colon cancers: A meta-analysis. Medicine.

[5] O’Sullivan D.E., Sutherland R.L., Town S., Chow K., Fan J., Forbes N., Heitman S.J., Hilsden R.J., Brenner D.R. (2022). Risk factors for early-onset colorectal cancer: A systematic review and meta-analysis. Clin. Gastroenterol. Hepatol..

[6] Révész D., Bours M.J., Wegdam J.A., Keulen E.T., Breukink S.O., Slooter G.D., Vogelaar F.J., Weijenberg M.P., Mols F. (2021). Associations between alcohol consumption and anxiety, depression, and health-related quality of life in colorectal cancer survivors. J. Cancer Surviv..

[7] Shrimahitha D., Mayowa A., Nisha M., Vinutna G., Kruthiga R. (2022). Colon cancer and obesity: A narrative review. Cureus.

[8] Balhareth A., Aldossary M.Y., McNamara D. (2019). Impact of physical activity and diet on colorectal cancer survivors’ quality of life: A systematic review. World J. Surg. Oncol..

[9] Yu J., Feng Q., Kim J.H., Zhu Y. (2022). Combined effect of healthy lifestyle factors and risks of colorectal adenoma, colorectal cancer, and colorectal cancer mortality: Systematic review and meta-analysis. Front. Oncol..

[10] Song M., Wu K., Meyerhardt J.A., Ogino S., Wang M., Fuchs C.S., Giovannucci E.L., Chan A.T. (2018). Fiber intake and survival after colorectal cancer diagnosis. JAMA Oncol..

[11] Ton M., Watson N.F., Sillah A., Malen R.C., Labadie J.D., Reedy A.M., Cohen S.A., Burnett-Hartman A.N., Newcomb P.A., Phipps A.I. (2021). Colorectal Cancer anatomical site and sleep quality. Cancers.

[12] Wele P., Wu X., Shi H. (2022). Sex-dependent differences in colorectal cancer: With a focus on obesity. Cells.

[13] Orive M., Anton-Ladislao A., Lázaro S., Gonzalez N., Bare M., Fernandez de Larrea N., Redondo M., Bilbao A., Sarasqueta C., Aguirre U. (2022). Anxiety, depression, health-related quality of life, and mortality among colorectal patients: 5-year follow-up. Support. Care Cancer.

[14] Ratjen I., Schafmayer C., Enderle J., di Giuseppe R., Waniek S., Koch M., Lieb W. (2018). Health-related quality of life in long-term survivors of colorectal cancer and its association with all-cause mortality: A German cohort study. BMC Cancer.

[15] McDougall J.A., Blair C.K., Wiggins C.L., Goodwin M.B., Chiu V.K., Rajput A., Kinney A.Y. (2019). Socioeconomic disparities in health-related quality of life among colorectal cancer survivors. J. Cancer Surviv..

[16] Moreno P.I., Ramirez A.G., San Miguel-Majors S.L., Fox R.S., Castillo L., Gallion K.J., Penedo F.J. (2018). Satisfaction with cancer care, self-efficacy, and health-related quality of life in Latino cancer survivors. Cancer.

[17] Charlson M.E., Pompei P., Ales K.L., MacKenzie C.R. (1987). A new method of classifying prognostic comorbidity in longitudinal studies: Development and validation. J. Chronic Dis..

[18] Craig C.L., Marshall A.L., Sjöström M., Bauman A.E., Booth M.L., Ainsworth B.E., Pratt M., Ekelund U., Yngve A., Sallis J.F. (2003). International Physical Activity Questionnaire: 12-country reliability and validity. Med. Sci. Sports Exerc..

[19] World Health Organization Physical Activity. https://www.who.int/news-room/fact-sheets/detail/physical-activity.

[20] Yang S.P., Wang T.J., Huang C.C., Chang S.C., Liang S.Y., Yu C.H. (2021). Influence of albumin and physical activity on postoperative recovery in patients with colorectal cancer: An observational study. Eur. J. Oncol. Nurs..

[21] Thompson F.E., Subar A.F., Smith A.F., Midthune D., Radimer K.L., Kahle L.L., Kipnis V. (2002). Fruit and vegetable assessment: Performance of 2 new short instruments and a food frequency questionnaire. J. Am. Diet. Assoc..

[22] Buysse D.J., Reynolds C.F., Monk T.H., Berman S.R., Kupfer D.J. (1989). The pittsburgh sleep quality index: A new instrument for psychiatric practice and research. Psychiatry Res..

[23] Cella D.F., Tulsky D.S., Gray G., Sarafian B., Linn E., Bonomi A., Silberman M., Yellen S.B., Winicour P., Brannon J. (1993). The Functional Assessment of Cancer Therapy scale: Development and validation of the general measure. J. Clin. Oncol..

[24] Yost K.J., Cella D., Chawla A., Holmgren E., Eton D.T., Ayanian J., West D.W. (2005). Minimally important differences were estimated for the functional assessment of cancer therapy–colorectal (FACT-C) instrument using a combination of distribution-and anchor-based approaches. Clin. Epidemiol..

[25] Ward W., Hahn E., Mo F., Hernandez L., Tulsky D., Cella D. (1999). Reliability and validity of the Functional Assessment of Cancer Therapy-Colorectal (FACT-C) quality of life instrument. Qual. Life Res..

[26] Wong C.K., Lam C.L., Rowen D., McGhee S.M., Ma K.P., Law W.L., Poon J.T., Chan P., Kwong D.L., Tsang J. (2012). Mapping the functional assessment of cancer therapy-general or-colorectal to SF-6D in Chinese patients with colorectal neoplasm. Value Health.

[27] Reudink M., Molenaar C.J.L., Bonhof C.S., Janssen L., Mols F., Slooter G.D. (2022). Evaluating the longitudinal effect of colorectal surgery on health-related quality of life in patients with colorectal cancer. J. Surg. Oncol..

[28] Valeikaite-Tauginiene G., Kraujelyte A., Poskus E., Jotautas V., Saladzinskas Z., Tamelis A., Lizdenis P., Dulskas A., Samalavicius N.E., Strupas K. (2022). Predictors of quality of life six years after curative colorectal cancer surgery: Results of the prospective multicenter study. Medicina.

[29] Al-Shandudi M., Al-Moundhri M., Chan M.F., Al-Hajri T., Al-Balushi M., Al-Azri M. (2022). Health-related quality of life, functioning, and physical symptoms of adult omani colorectal cancer survivors. Asian Pac. J. Cancer Prev..

[30] Kennedy F., Lally P., Miller N.E., Conway R.E., Roberts A., Croker H., Fisher A., Beeken R.J. (2023). Fatigue, quality of life and associations with adherence to the World Cancer Research Fund guidelines for health behaviours in 5835 adults living with and beyond breast, prostate and colorectal cancer in England: A cross-sectional study. Cancer Med..

[31] Trudel-Fitzgerald C., Tworoger S.S., Poole E.M., Zhang X., Giovannucci E.L., Meyerhardt J.A., Kubzansky L.D. (2018). Psychological symptoms and subsequent healthy lifestyle after a colorectal cancer diagnosis. Health Psychol..

[32] Breedveld-Peters J.J.L., Koole J.L., Muller-Schulte E., van der Linden B.W.A., Windhausen C., Bours M.J.L., van Roekel E.H., Weijenberg M.P. (2018). Colorectal cancers survivors’ adherence to lifestyle recommendations and cross-sectional associations with health-related quality of life. Br. J. Nutr..

[33] Ocalewski J., Jankowski M., Zegarski W., Migdalski A., Buczkowski K. (2023). the role of health behaviors in quality of life: A longitudinal study of patients with colorectal cancer. Int. J. Environ. Res. Public Health.

[34] Tiselius C., Rosenblad A., Strand E., Smedh K. (2021). Risk factors for poor health-related quality of life in patients with colon cancer include stoma and smoking habits. Health Qual. Life Outcomes.

[35] Brown J.C., Damjanov N., Courneya K.S., Troxel A.B., Zemel B.S., Rickels M.R., Ky B., Rhim A.D., Rustgi A.K., Schmitz K.H. (2018). A randomized dose-response trial of aerobic exercise and health-related quality of life in colon cancer survivors. Psycho-Oncol..

[36] Eyl R.E., Xie K., Koch-Gallenkamp L., Brenner H., Arndt V. (2018). Quality of life and physical activity in long-term (≥5 years post-diagnosis) colorectal cancer survivors—Systematic review. Health Qual. Life Outcomes.

[37] Rodriguez J.L., Hawkins N.A., Berkowitz Z., Li C. (2015). Factors associated with health-related quality of life among colorectal cancer survivors. Am. J. Prev. Med..

[38] Kenkhuis M.F., van Duijnhoven F.J., van Roekel E.H., Breedveld-Peters J.J., Breukink S.O., Janssen-Heijnen M.L., Keulen E.T.P., Mols F., Weijenberg M.P., Bours M.J. (2022). Longitudinal associations of fiber, vegetable, and fruit intake with quality of life and fatigue in colorectal cancer survivors up to 24 months posttreatment. Am. J. Clin. Nutr..

[39] Van Veen M.R., Mols F., Bours M.J., Weijenberg M.P., Kampman E., Beijer S. (2019). Adherence to the World Cancer Research Fund/American Institute for Cancer Research recommendations for cancer prevention is associated with better health–related quality of life among long-term colorectal cancer survivors: Results of the PROFILES registry. Support. Care Cancer.

